# Efficacy and safety of massage in the treatment of post-stroke insomnia: A protocol for systematic review and meta-analysis

**DOI:** 10.1097/MD.0000000000023598

**Published:** 2020-12-18

**Authors:** Yajing Zhang, Xingwei He, Shasha Hu, Songfeng Hu, Fan He, Yu Shen, Fenfen Zhao, Qin Zhang, Tingping Liu, Changkang Wang

**Affiliations:** a College of Acupuncture-Moxibustion and Tuina, Jiangxi University of Traditional Chinese Medicine; b The Affiliated Hospital of Jiangxi University of Traditional Chinese Medicine; c The Second Affiliated Hospital of Nanchang University, Nanchang, Jiangxi, China.

**Keywords:** massage, post-stroke insomnia, protocol, systematic review

## Abstract

**Background:**

: Post-stroke insomnia (PSI) is a serious problem which has significant adverse effects on the subsequent recovery of patients and the quality of their daily life. Massage is effective in improving the quality of sleep for stroke patients displaying no significant adverse reactions. Up to now, however, there are still no systematic studies conducted to provide compelling evidence for its effectiveness in treating PSI. Allowing for this, this project is purposed to make a thorough summary of the efficacy of massage therapy in treating PSI and the safety of this practice.

**Methods:**

: Without considering the status of publication and language, a meticulous search will be conducted, covering the Web of Science, the Cochrane Library search, PubMed, EMBASE, Chinese biomedical literature database, Chongqing VIP Database for Chinese Technical Periodicals, China National Knowledge Infrastructure, and Wanfang. All randomized controlled trials of PSI will be retrieved. The deadline is set as October 23, 2020. The team will be comprised of 2 experienced researchers who will apply RevMan V.5.3 software to conduct literature selection, data collection, data analysis, and data synthesis, respectively. In addition, the Cochrane risk Assessment tool will be taken as the top choice to evaluate the quality of the trials involved in this study.

**Results:**

: The effectiveness and safety of massage therapy intended for PSI will be subject to a systematic evaluation under this program.

**Conclusion:**

: It will be substantiated in this review whether massage therapy is a reliable intervention for PSI by examining the evidence collected.

## 1 Introduction

As an acute cerebrovascular disease, stroke is a leading cause of fatality and disability among adults. China has the highest incidence of stroke worldwide.^[1]^ Insomnia is a common problem caused by stroke. As revealed by meta-analysis, the incidence rate of PSI was approximately 38.2%.^[2]^ To be specific, post-stroke insomnia (PSI) is often manifested as the difficulty in falling asleep, the ease to wake up while asleep or the declining quality of sleep. If left untreated, it may cause such problems as anxiety, depression, cognitive impairment, and mental disorder^[3]^; also can concomitant endocrine, cardiovascular and other diseases^[4]^; and an analysis of the study suggested that insomnia increases the risk of ischemic stroke.^[5]^ Therefore, the treatment of PSI is of particular importance.

Currently, drug therapy is the major treatment for PSI. The American Academy of Sleep Medicine, The American College of Physicians, and the British Psychopharmacology Association recommended that gamma-aminobutyric acid_A_ receptor agonists, including benzodiazepines and non-benzodiazepines hypnotics, and selective melatonin receptor agonists (MT1 and MT2) to be first line of treatment for insomnia.^[6–9]^ These drugs have an important therapeutic effect on PSI.^[10]^ However, attention shall be paid to a range of side effects such as nausea, anxiety, drowsiness, mental confusion, and drug dependence.^[11–12]^ Apart from that, these drugs have a potential to affect nerve repair in stroke patients.^[11]^ In addition, these drugs are expensive and not affordable for the general population in the long term.^[10]^ To sum up, it is imperative to find a treatment intervention that is cheaper, safer yet less likely to produce side effects for those patients suffering from PSI. According to studies, massage is capable to significantly improve the symptoms of insomniacs, create a pleasant feeling, and reduce the occurrence of significant adverse events.^[13–17]^

As a means of medical treatment, massage is a widely used complementary and alternative therapy.^[15]^ First of all, massage is conducive to promoting blood circulation and enhancing immunity, thus achieving the purpose of preventing and treating diseases. Second, it has been demonstrated in existing studies that the comfort generated by localized massaging can be transmitted to the central nervous system through peripheral receptors, which leads to a feeling of relaxation, tranquility, and calmness.^[16–19]^ Finally, massage therapy has the advantages of simple operation, no obvious adverse reactions, and low price. So far, a number of studies have revealed that massage is effective in alleviating the symptoms associated with insomnia.^[14,18–21]^

To the best of our knowledge, however, there has yet to be any systematic review conducted of whether massage therapy is safe and effective in treating PSI. Therefore, this program is aimed at evaluating the effectiveness of massage in treating PSI in a comprehensive way.

## 2 Methods

### 2.1 Study registration

The registration number is INPLASY2020100113. This agreement will be applied in strict accordance with the preferred reporting items of the Protocol for Systematic Review and Meta-Analysis Statement Guidelines.^[22]^

### 2.2 Inclusion criteria for study

#### 2.2.1 Type of studies

In addition to individual case reports, review, the summaries of experience, animal studies, and non-randomized controlled trials (RCTs), this study will include all RCTs of massage therapy in patients with PSI, regardless of language or publication status.

#### 2.2.2 Types of participants

All patients diagnosed with PSI were included, and there were no limitations on gender, age, or race.

#### 2.2.3 Types of interventions

##### 2.2.3.1 Experimental interventions

The experimental group was restricted to receiving massage therapy, including reflex, finger point, foot massage, whole body massage, and so on. Besides, there was no restriction applied to the frequency, intensity, duration, location, and type of massage therapy.

##### 2.2.3.2 Control interventions

As for the control group, drug therapy, cognitive intervention therapy, phototherapy, aromatherapy, music therapy, acupuncture, psychological therapy, foot bath therapy, and other means of treatment than massage were selected.

#### 2.2.4 Types of outcome measures

##### 2.2.4.1 Primary outcomes

Pittsburgh sleep quality index

##### 2.2.4.2 Secondary outcomes

The following elements will serve as secondary results:

1. Adverse events
2. Athens insomnia scale
3. The insomnia severity index

### 2.3 Search methods

#### 2.3.1 The primary source of data

The scope of search includes the Chinese biomedical literature database, Chongqing VIP Database for Chinese Technical Periodicals, China National Knowledge Infrastructure, Wanfang, Web of Science, Cochrane Library, PubMed, EMBASE, and other databases, with the deadline of October 23, 2020. Besides, the literature on massage intervention will be searched for PSI. In addition, we will be working from the China Clinical trials Registry and clinicaltrials.gov. retrieve some uncompleted or unpublished trial data. Table 1 details the retrieval strategies adopted by PubMed.

**Table 1:** Search strategy for PubMed database.

Number	Search items
#1	randomized controlled trial [pt]
#2	controlled clinical trial [pt]
#3	randomized [tiab]
#4	clinical trials as topic [mesh: noexp]
#5	randomly [tiab]
#6	trial [ti]
#7	OR/#1–#6
#8	animals [mh] NOT humans [mh]
#9	#7 NOT #8
#10	Stroke[Mesh]
#11	Cerebral Stroke[All Fields)
#12	Apoplexy[All Fields)
#13	Cerebrovascular Apoplexy[All Fields)
#14	Cerebrovascular Accident[All Fields)
#15	OR/#10–#14
#16	Insomnia[Mesh]
#17	Insomnias[All Fields)
#18	Awakening[All Fields)
#19	Sleeplessness[All Fields)
#20	Chronic Insomnia[All Fields)
#21	OR/#16–#20
#22	Massage[Mesh]
#23	Massage Therapy[All Fields)
#24	Zone Therapy[All Fields)
#25	Acupressure[All Fields)
#26	Manipulate[All Fields)
#27	Tuina[All Fields)
#28	Anmo[All Fields)
#29	OR/#21–#27
#30	#9 AND #15 AND #21AND #28

### 2.4 Data collection and analysis

#### 2.4.1 Literature selection

**Figure 1:**
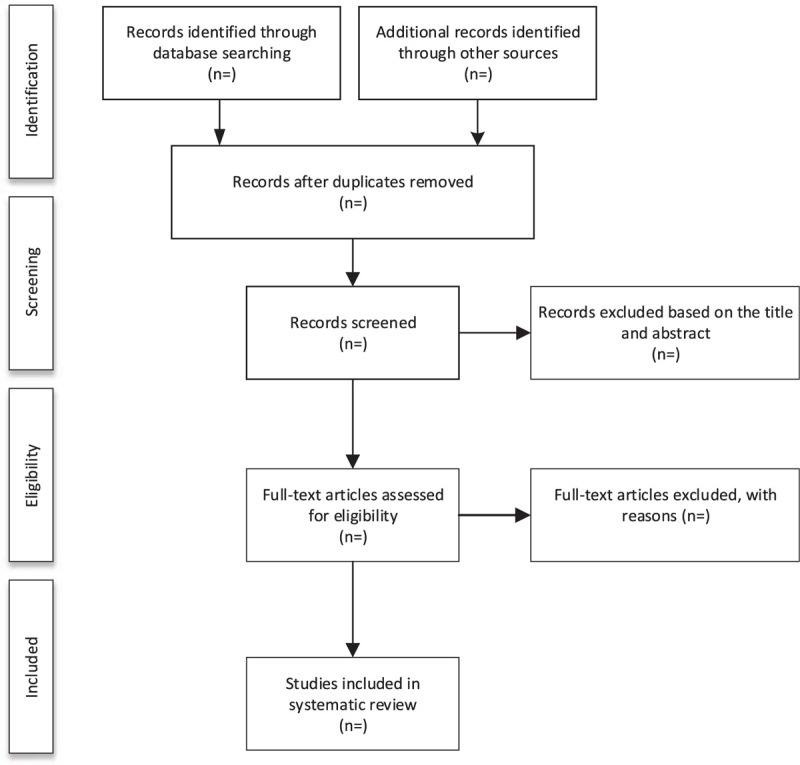
Flow diagram of study selection process.

First of all, all literature will be inputted into EndNote X9 and all duplicate literature will be removed. Second, researchers SQ and YX will read the titles and abstracts separately before screening them to remove irrelevant literature. Third, they carefully read the full text to determine whether the literature is eligible to be included in the study. Finally, 2 researchers (SQ and YX) will conduct cross-check. In case of any disagreement, a third researcher (LJ) will get involved in the discussion to solve it. Figure 1 shows the process of literature screening.

#### 2.4.2 Data extraction and management

Two researchers extracted the following data using a data record table prepared:

1. Journal, the information about the author, title, the time of publication;
2. The characteristics of participants, sample size, intervention measures, and the severity of insomnia;
3. Research methods: Randomization, allocation concealment, blinding, result analysis
4. Primary and secondary outcome measurements as well as any adverse events.

### 2.5 Risk of bias assessment

In order to assess the quality of relevant trials, SQ and YX will apply the Cochrane Bias risk Assessment tool,^[22]^ respectively. The extracted details include the random sequence generation, the blindness of result evaluation, the blindness of participants and personnel, the concealment of allocation, the reporting of selective results, the incomplete result data, and so on. In the meantime, they are divided into 3 different levels: fuzzy, low, and high. When the relevant information to research project is found unavailable, contact will be made with the author of the project to obtain the required information. In case of any dispute, a more sensible decision will be made with the assistance of the Third investigator (LJ).

### 2.6 Data synthesis

RevMan5.3 software was applied to conduct data analysis. Relative risk was treated as the effect analysis statistic for binary variables. Mean difference was taken as the effect analysis system for continuous variables, and 95% confidence interval was adopted to carry out interval estimation. The heterogeneity between the results using Chi-square test analysis (alpha test level = 0.1), and combining with quantitative judgment *I*^2^ heterogeneity is big is small, if *I*^2^ ≤ 50%, *P* ≥ .1, show good homogeneity between the various research, using the fixed effects model, if not that the statistical heterogeneity between the results of the study is larger, should further analyze sources of heterogeneity, and use the random effects model. If the level of clinical heterogeneity is significant, sensitivity analysis will be conducted, otherwise only descriptive analysis will be carried out.

### 2.7 Management of missing data

If the data used in the study lacks clarity or is unavailable, the author of the article will be contacted either on telephone or by email to obtain a data set that is as complete as possible. If the data needed is still not obtained, only the existing data will be used for analysis.

### 2.8 Subgroup analysis

In case of any significant heterogeneity among the tests involved, consideration will be given to the subgroup analysis of types of stroke, massage mode, the severity of insomnia, the progression of disease, sample size, and other factors.

### 2.9 Sensitivity analysis.

In order to ensure the stability and reliability of the conclusions drawn from the meta-analysis, sensitivity analysis will be conducted to remove low-quality literature.

### 2.10 Assessment of reporting biases

If the number of RCTs exceeds 10, funnel plot analysis will be required for publication bias test. In addition, in case of an asymmetric funnel graph, Egger test will be conducted to find out about the causes of publication bias.

### 2.11 Quality of evidence

The quality of evidence assessment will be measured by 2 researchers using Grade Profiler 3.6 software. As for the assessment scale, it is comprised of high evidence, intermediate evidence, low evidence, and very low evidence.^[23]^

### 2.12 Ethics and dissemination

As this study is irrelevant to the personal details of patients, it is exempt from ethical approval. The results of the study are expected to be published in a peer-reviewed journal.

## 3 Discussion

PSI is frequently manifested as the difficulty in falling asleep, the ease to wake up while asleep, and the poor quality of sleep, which can not only affect the later-stage recovery of stroke patients, but also increase the risk of recurring stroke.^[1–3]^ Currently, drug therapy is regarded as the major effective solution to treating PSI. However, it is also possible for drug therapy to produce a variety of side effects, and medication is expensive. As a common adjunct therapy, massage is effective in alleviating the symptoms associated with insomnia, thus improving the quality of life for stroke patients.^[18–21]^ In addition, it demonstrates such advantages as cheap affordability, high safety, the ease of operation, and limited side effects, which makes massage therapy an ideal choice for the patients suffering PSI. Up to now, the efficacy of massage therapy in treating PSI remains to be substantiated. Therefore, it is hoped that this study can provide some credible evidence and valuable medical reference for the treatment of PSI through massage therapy.

However, this study may have some limitations. First, there may be heterogeneity due to different massage methods. Second, this study does not restrict language, which may lead to language bias.

## Author contributions

**Conceptualization:** Yajing Zhang, Xingwei He.

**Data curation:** Yajing Zhang, Shasha Hu.

**Formal analysis:** Yajing Zhang, Xingwei He.

**Funding acquisition:** Xingwei He.

**Methodology:** Yajing Zhang, Shasha Hu, Yu Shen.

**Software:** Yajing Zhang, Shasha Hu.

**Supervision:** Xingwei He.

**Writing – original draft:** Yajing Zhang, Xingwei He.

**Writing – review and editing:** Xingwei He.
